# Ball bearing vibration data for detecting and quantifying spall faults

**DOI:** 10.1016/j.dib.2023.109019

**Published:** 2023-03-01

**Authors:** Mohamed A.A. Ismail, Jens Windelberg, Andreas Bierig, Iñaki Bravo, Aitor Arnaiz

**Affiliations:** a German Aerospace Center (DLR) - Institute of Flight Systems, Lilienthalplatz 7, 38108 Braunschweig, Germany; b Intelligent Information Systems, TEKNIKER, Eibar, 20600, Spain

**Keywords:** Bearing defects, Condition monitoring, Health assessment, Predictive maintenance

## Abstract

Ball bearings are essential components of electromechanical systems, and their failures significantly affect the service lifetime of these systems. For highly reliable and safety-critical electromechanical systems in energy and aerospace sectors, early bearing fault detection and quantification are crucial. The vibration measurements of bearing fatigue faults, i.e., spalls, are typically induced by multiple excitation mechanisms depending on the fault size and the operating conditions. This data article contains vibration datasets for faulty ball bearings, including the common vibration excitation mechanisms for various fault sizes and operating conditions. These faults are artificially seeded on bearing races by a precise machining process to emulate realistic fatigue faults. This data article is beneficial for better understanding the vibration signal characteristics under different fault sizes and for validating condition monitoring methods for various industrial and aerospace applications.

**Specifications Table**

**Table:** 

Subject	Mechanical Engineering
Specific subject area	Vibration-Based Condition Monitoring
Type of data	Datasets in “.mat” format Figures
How the data were acquired	A test setup based on FALEX multispecimen test bench and data acquisition system, comprising an accelerometer (model PCB 356A32), an axial loading mechanism (up to 8.8 kN) and a closed control for the bearing spindle. The data is based on a common aerospace bearing model FAG QJ212TVP. The bearing under test was fitted vertically in the test bench where a vertical axial load was applied to the inner race to match high loading at low speed actuation conditions in aerospace and energy systems.
Data format	Raw
Description of data collection	There are 28 datasets for ball bearings with different artificial fault sizes, sampling rate of 25.6 kHz and four operational factors: the rotational speed, the applied axial load, the fault size and the fault location. The key measurement criterion is to keep large number of data samples per fault size to study how the fault size and shape influence the instantaneous characteristics of vibration measurements. The fault type was a fatigue spall defect on the inner or outer race. The full 3D geometry of every fault is defined in the data description section. The faults were seeded to the races by spark-erosion machining to physically emulate real spall defects.
Data source location	DLR (German Aerospace Center) Institute of Flight Systems Condition Monitoring Research Group Braunschweig, Germany
Data accessibility	Repository name: Vibration Data for Axial Ball Bearings and Spall Faults, (Mendeley Data) Data identification number (doi):10.17632/chwhh9n3bf.2 Direct URL to data: http://dx.doi.org/10.17632/chwhh9n3bf.2
Related research article	M.A.A. Ismail, A. Bierig, N. Sawalhi, Automated vibration-based fault size estimation for ball bearings using Savitzky–Golay differentiators, J. Vib. Control. 24(18) (2018) 4297–4315. https://doi.org/10.1177/1077546317723227.

## Value of the Data

• These datasets are acquired under various bearing fault sizes at low rotational speeds and a high sampling rate. These conditions help understand how the fault geometry influences the instantaneous characteristics of vibration measurements.
• These datasets can be used to study the time and frequency features of vibration measurements for healthy and spalled axial bearings.
• These datasets are beneficial as a benchmark for developing fault size quantification and prognosis methods for various industrial and aerospace applications.

## 1 Objective

This data article is designed to support the fault size estimation research of ball bearings. The knowledge generated can be utilized for developing and validating efficient vibration-based predictive maintenance for energy and aerospace systems. The datasets are measured under various bearing fault sizes at low rotational speeds and a high sampling rate. These conditions aimed at guaranteed large number of data samples per fault-width (along the bearing's rotational speed) to study how the fault geometry (fault size and shape) influences the instantaneous characteristics of vibration measurements [1]. The gained knowledge was utilized to build a reliable vibration-based fault size estimation method without using machine learning [2]. It was observed that different fault sizes induce significant time transients that can be detected by the first time derivative of the vibration jerk better than the raw acceleration signal. This observation was used to extract the fault entry/exit instants (i.e. the fault width) from the vibrational jerk, which were numerically estimated from raw accelerometer data using Savitzky–Golay differentiators. Serviceable fault estimation for different operating speeds and load levels were successfully achieved for 91% of the datasets with a maximum estimation error of 20%.

## 2 Data Description

**Fig. 1:**
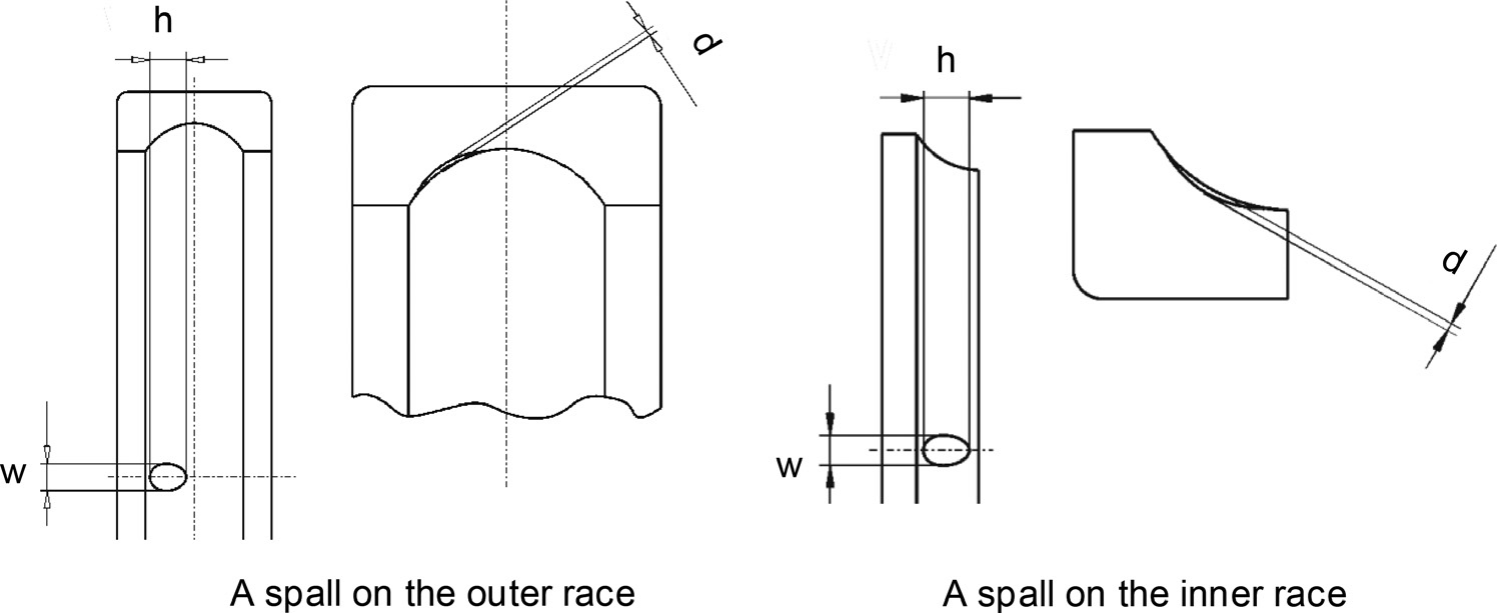
Geometric profiles of outer and inner race spalls

**Fig. 2:**
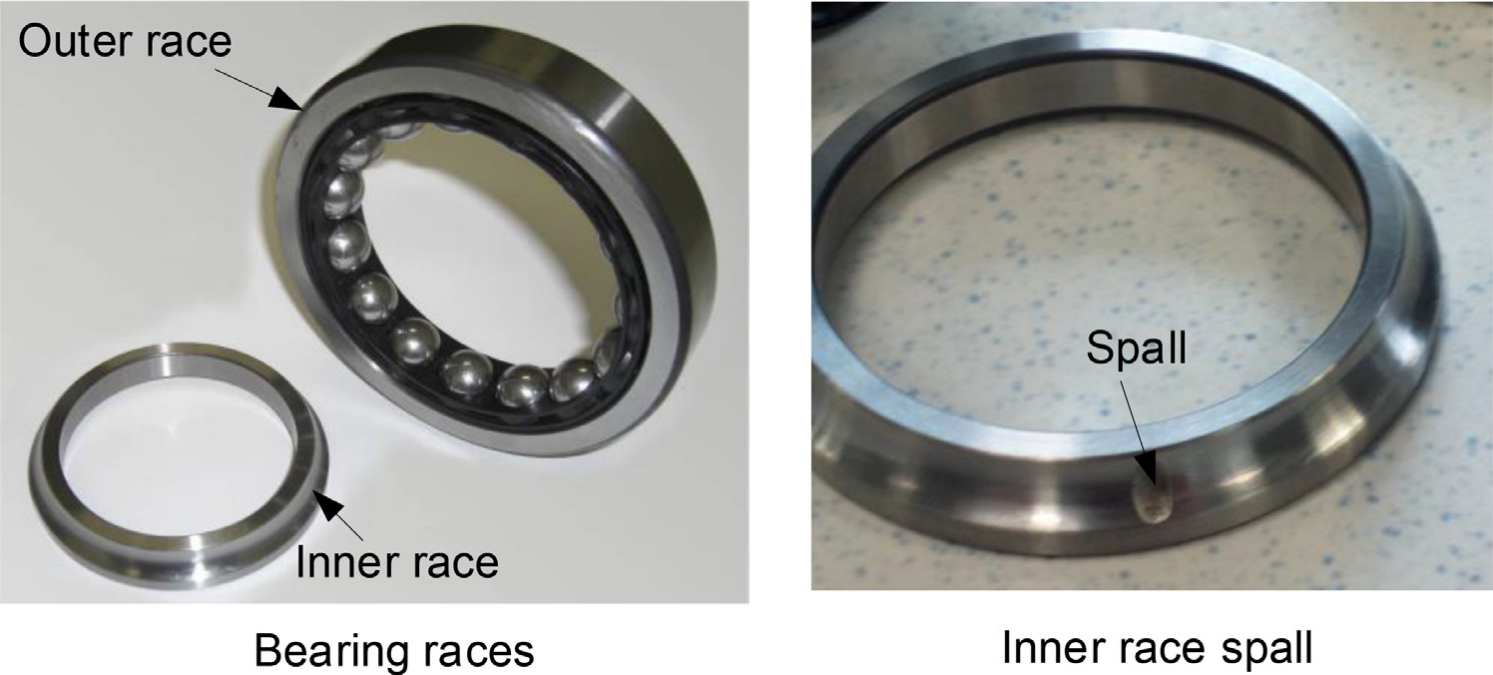
Photographs of QJ212TVP bearing races and a seeded inner race spall

The datasets include 28 time series vibration measurements and they are available in [1]. They were used for vibration-based fault detection and quantification in previous research [2,3]. These datasets were collected under healthy and faulty conditions based on the common aerospace bearing model FAG QJ212TVP [4]. The fault type was a fatigue spall on the inner or outer race. These faults were created by spark-erosion machining. As shown in Figs. 1 and 2, the geometry of a spall fault was identified by three parameters: width w, height h, and depth d. Each dataset was measured with a sampling frequency of 25.6 kHz. The datasets were stored in the standard MATLAB format, “.mat,” in a single column without a time stamp, and they were collected at constant speeds for a fixed duration of 30 s.

The descriptions of the datasets according to the health and the operating conditions are provided as follows:

Healthy bearings group:

1. N5k_60_0.0.mat: a vibration dataset for a healthy bearing. The outer race is fixed and the inner race is connected to a spindle operating at 60 RPM and 5.0 kN axial load.
2. N8.8k_60_0.0.mat: a vibration dataset for a healthy bearing. The outer race is fixed and the inner race is connected to a spindle operating at 60 RPM and 8.8 kN axial load.
3. N5k_500_0.0.mat: a vibration dataset for a healthy bearing. The outer race is fixed and the inner race is connected to a spindle operating at 500 RPM and 5.0 kN axial load.
4. N8.8k_500_0.0.mat: a vibration dataset for a healthy bearing. The outer race is fixed and the inner race is connected to a spindle operating at 500 RPM and 8.8 kN axial load.

Faulty bearings group:

1. N5k_60_1.0_inner.mat: a vibration dataset for a faulty bearing on the inner race. The fault dimensions are: w = 1.0 mm, d = 0.05 mm, h = 2.6 mm as shown in Fig 1. The outer race is fixed and the inner race is connected to a spindle operating at 60 RPM and 5.0 kN axial load.
2. N5k_60_2.1_inner.mat: a vibration dataset for a faulty bearing on the inner race. The fault dimensions are: w = 2.1 mm, d = 0.20 mm, h = 5.0 mm as shown in Fig 1. The outer race is fixed and the inner race is connected to a spindle operating at 60 RPM and 5.0 kN axial load.
3. N5k_60_3.8_inner.mat: a vibration dataset for a faulty bearing on the inner race. The fault dimensions are: w = 3.8 mm, d = 0.40 mm, h = 6.8 mm as shown in Fig 1. The outer race is fixed and the inner race is connected to a spindle operating at 60 RPM and 5.0 kN axial load.
4. N5k_500_1.0_inner.mat: a vibration dataset for a faulty bearing on the inner race. The fault dimensions are: w = 1.0 mm, d = 0.05 mm, h = 2.6 mm as shown in Fig 1. The outer race is fixed and the inner race is connected to a spindle operating at 500 RPM and 5.0 kN axial load.
5. N5k_500_2.1_inner.mat: a vibration dataset for a faulty bearing on the inner race. The fault dimensions are: w = 2.1 mm, d = 0.20 mm, h = 5.0 mm as shown in Fig 1. The outer race is fixed and the inner race is connected to a spindle operating at 500 RPM and 5.0 kN axial load.
6. N5k_500_3.8_inner.mat: a vibration dataset for a faulty bearing on the inner race. The fault dimensions are: w = 3.8 mm, d = 0.40 mm, h = 6.8 mm as shown in Fig 1. The outer race is fixed and the inner race is connected to a spindle operating at 500 RPM and 5.0 kN axial load.
7. N8.8k_60_1.0_inner.mat: a vibration dataset for a faulty bearing on the inner race. The fault dimensions are: w = 1.0 mm, d = 0.05 mm, h = 2.6 mm as shown in Fig 1. The outer race is fixed and the inner race is connected to a spindle operating at 60 RPM and 8.8 kN axial load.
8. N8.8k_60_2.1_inner.mat: a vibration dataset for a faulty bearing on the inner race. The fault dimensions are: w = 2.1 mm, d = 0.20 mm, h = 5.0 mm as shown in Fig 1. The outer race is fixed and the inner race is connected to a spindle operating at 60 RPM and 8.8 kN axial load.
9. N8.8k_60_3.8_inner.mat: a vibration dataset for a faulty bearing on the inner race. The fault dimensions are: w = 3.8 mm, d = 0.40 mm, h = 6.8 mm as shown in Fig 1. The outer race is fixed and the inner race is connected to a spindle operating at 60 RPM and 8.8 kN axial load.
10. N8.8k_500_1.0_inner.mat: a vibration dataset for a faulty bearing on the inner race. The fault dimensions are: w = 1.0 mm, d = 0.05 mm, h = 2.6 mm as shown in Fig 1. The outer race is fixed and the inner race is connected to a spindle operating at 500 RPM and 8.8 kN axial load.
11. N8.8k_500_2.1_inner.mat: a vibration dataset for a faulty bearing on the inner race. The fault dimensions are: w = 2.1 mm, d = 0.20 mm, h = 5.0 mm as shown in Fig 1. The outer race is fixed and the inner race is connected to a spindle operating at 500 RPM and 8.8 kN axial load.
12. N8.8k_500_3.8_inner.mat: a vibration dataset for a faulty bearing on the inner race. The fault dimensions are: w = 3.8 mm, d = 0.40 mm, h = 6.8 mm as shown in Fig 1. The outer race is fixed and the inner race is connected to a spindle operating at 500 RPM and 8.8 kN axial load.
13. N5k_60_1.4_outer.mat: a vibration dataset for a faulty bearing on the outer race. The fault dimensions are: w = 1.4 mm, d = 0.05 mm, h = 2.6 mm as shown in Fig 1. The outer race is fixed and the inner race is connected to a spindle operating at 60 RPM and 5.0 kN axial load.
14. N5k_60_2.4_outer.mat: a vibration dataset for a faulty bearing on the outer race. The fault dimensions are: w = 2.4 mm, d = 0.20 mm, h = 5.0 mm as shown in Fig 1. The outer race is fixed and the inner race is connected to a spindle operating at 60 RPM and 5.0 kN axial load.
15. N5k_60_4.0_outer.mat: a vibration dataset for a faulty bearing on the outer race. The fault dimensions are: w = 4.0 mm, d = 0.40 mm, h = 6.8 mm as shown in Fig 1. The outer race is fixed and the inner race is connected to a spindle operating at 60 RPM and 5.0 kN axial load.
16. N5k_500_1.4_outer.mat: a vibration dataset for a faulty bearing on the outer race. The fault dimensions are: w = 1.4 mm, d = 0.05 mm, h = 2.6 mm as shown in Fig 1. The outer race is fixed and the inner race is connected to a spindle operating at 500 RPM and 5.0 kN axial load.
17. N5k_500_2.4_outer.mat: a vibration dataset for a faulty bearing on the outer race. The fault dimensions are: w = 2.4 mm, d = 0.20 mm, h = 5.0 mm as shown in Fig 1. The outer race is fixed and the inner race is connected to a spindle operating at 500 RPM and 5.0 kN axial load.
18. N5k_500_4.0_outer.mat: a vibration dataset for a faulty bearing on the outer race. The fault dimensions are: w = 4.0 mm, d = 0.40 mm, h = 6.8 mm as shown in Fig 1. The outer race is fixed and the inner race is connected to a spindle operating at 500 RPM and 5.0 kN axial load.
19. N8.8k_60_1.4_outer.mat: a vibration dataset for a faulty bearing on the outer race. The fault dimensions are: w = 1.4 mm, d = 0.05 mm, h = 2.6 mm as shown in Fig 1. The outer race is fixed and the inner race is connected to a spindle operating at 60 RPM and 8.8 kN axial load.
20. N8.8k_60_2.4_outer.mat: a vibration dataset for a faulty bearing on the outer race. The fault dimensions are: w = 2.4 mm, d = 0.20 mm, h = 5.0 mm as shown in Fig 1. The outer race is fixed and the inner race is connected to a spindle operating at 60 RPM and 8.8 kN axial load.
21. N8.8k_60_4.0_outer.mat: a vibration dataset for a faulty bearing on the outer race. The fault dimensions are: w = 4.0 mm, d = 0.40 mm, h = 6.8 mm as shown in Fig 1. The outer race is fixed and the inner race is connected to a spindle operating at 60 RPM and 8.8 kN axial load.
22. N8.8k_500_1.4_outer.mat: a vibration dataset for a faulty bearing on the outer race. The fault dimensions are: w = 1.4 mm, d = 0.05 mm, h = 2.6 mm as shown in Fig 1. The outer race is fixed and the inner race is connected to a spindle operating at 500 RPM and 8.8 kN axial load.
23. N8.8k_500_2.4_outer.mat: a vibration dataset for a faulty bearing on the outer race. The fault dimensions are: w = 2.4 mm, d = 0.20 mm, h = 5.0 mm as shown in Fig 1. The outer race is fixed and the inner race is connected to a spindle operating at 500 RPM and 8.8 kN axial load.
24. N8.8k_500_4.0_outer.mat: a vibration dataset for a faulty bearing on the outer race. The fault dimensions are: w = 4.0 mm, d = 0.40 mm, h = 6.8 mm as shown in Fig 1. The outer race is fixed and the inner race is connected to a spindle operating at 500 RPM and 8.8 kN axial load.

## 3 Experimental Design, Materials and Methods

**Fig. 3:**
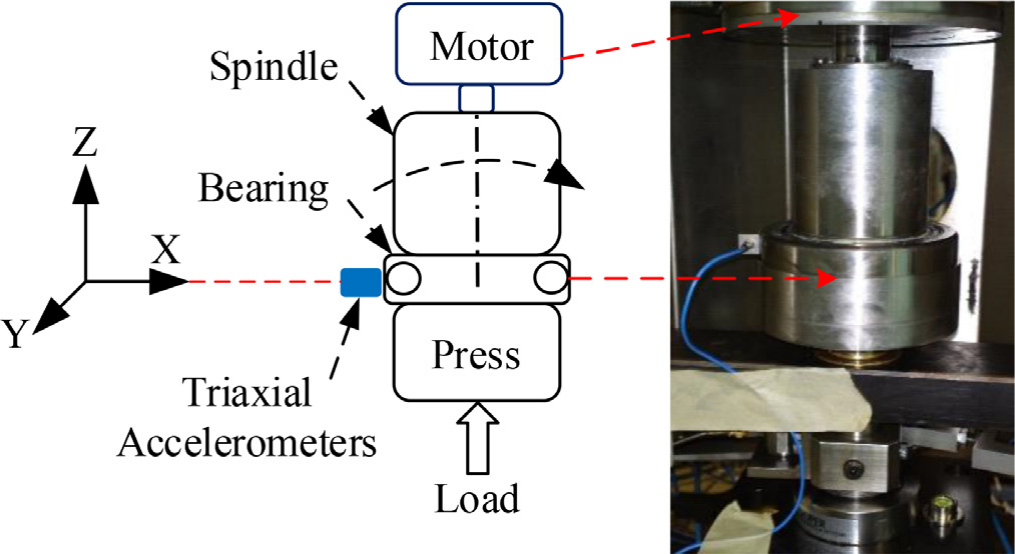
Schematic and photograph of a special bearing testing machine.

The experimentation of the bearings was performed in FALEX multispecimen test bench [5] available in Tekniker lab [6]. While the bearing faults have been designed and manufactured at DLR lab. The FALEX test bench was modified by adding a complete monitoring infrastructure, including force, speed, temperature and vibration sensors and a data acquisition system. The bearing test rig (Fig. 3) with three accelerometers, model PCB 356A32 [7], was used to measure triaxial vibrations along the x-, y-, and z-axes at a sampling frequency of 25.6 kHz [8]. The shared datasets include only x-axis measurements. The bearing under test was fitted vertically in the test rig, and a vertical axial load was applied to the inner race to emulate loading and speed conditions for electromechanical aerospace systems.

The datasets were collected and processed under four operating conditions: two speeds (60 RPM and 500 RPM) and two axial loads (5 kN and 8.8 kN). Table 1 lists the fault characteristic frequencies of the bearing for further signal processing for condition monitoring research.

**Table 1:** Bearing size data and fault characteristic frequencies for bearing QJ212TVP.

Parameter	Value
Number of balls	15
Ball diameter	15.87 mm
Ball pass frequency inner race*	8.6427 Hz
Ball pass frequency outer race*	6.3573 Hz
Bearing pitch diameter	85.15 mm

⁎ Estimated at a rotational speed of 1 Hz.

## Ethics Statements

This work does not involve human subjects, animal experiments, or any data collected from social media platforms,

## CRediT Author Statement

**Mohamed AA Ismail:** Data curation, Writing – original draft preparation; **Jens Windelberg:** Reviewing & editing; **Andreas Bierig:** Reviewing & Supervision; **Iñaki Bravo:** Data generation, curation and Reviewing. **Aitor Arnaiz**: Reviewing and Supervision.

## Declaration of Competing Interest

The authors declare that they have no known competing financial interests or personal relationships that could have appeared to influence the work reported in this paper.

## Data Availability

Vibration Data for Axial Ball Bearings and Spall Faults (Original data) (Mendeley Data).
